# Metabolism of strawberry mono- and dimeric ellagitannins in rats fed a diet containing fructo-oligosaccharides

**DOI:** 10.1007/s00394-015-1133-5

**Published:** 2015-12-21

**Authors:** Adam Jurgoński, Jerzy Juśkiewicz, Bartosz Fotschki, Krzysztof Kołodziejczyk, Joanna Milala, Monika Kosmala, Katarzyna Grzelak-Błaszczyk, Lidia Markiewicz

**Affiliations:** 1 Division of Food Science, Institute of Animal Reproduction and Food Research, Polish Academy of Sciences, Tuwima 10, 10-748 Olsztyn, Poland; 2 Institute of Food Technology and Analysis, Lódź University of Technology, Stefanowskiego 4/10, 90-924 Lodz, Poland

**Keywords:** Bifidobacteria, Fructans, Hydrolysable tannins, Lactic acid bacteria, Nasutins, Urolithins

## Abstract

**Purpose:**

We investigated the effects of dietary supplementation with strawberry extracts rich in ETs and fructo-oligosaccharides (FOS) on the intestinal microbiota and the formation of bacterial metabolites in the distal intestine, as well as the absorption of ET metabolites and antioxidant status in rats.

**Methods:**

Rats were allocated into six groups of eight animals each and fed for 4 weeks with a control diet (group C), a control diet supplemented with FOS (group C + FOS) or modifications of these diets, in which a monomeric or dimeric ET-rich extract was added (groups ME and ME + FOS or DE and DE + FOS, respectively).

**Results:**

The extract addition, the FOS addition and their interaction significantly affected the total and selected bacterial counts in the caecal digesta (all *P* < 0.005). The total bacterial count was the highest in group C + FOS, lower in group DE and the lowest in group ME + FOS (10.6, 10.3 and 8.52 log cells/g, respectively; *P* ≤ 0.05). The total caecal content of ET metabolites was higher in the ME and ME + FOS group than in the DE and DE + FOS group, respectively (67.8 and 89.5 vs. 13.0 and 18.0 µg/g, respectively; *P* < 0.001). The total plasma concentration of ET metabolites was higher in the ME + FOS and DE + FOS group than in the ME group (248 and 281 vs. 8.13 ng/mL, respectively; *P* < 0.001).

**Conclusions:**

ETs of the monomeric ET-rich extract are more prone to intestinal breakdown than those of the dimeric ET-rich extract, and absorption of their metabolites can be increased by dietary FOS; however, together, they evoke strong antibacterial activity.

## Introduction

Ellagitannins (ETs) are a heterogeneous class of plant polyphenols with a complex chemical structure, in which one or more groups of hexahydroxydiyphenic acid, an ellagic acid precursor, are linked to a central glycosidic unit, usually glucose [1, 2]. These diverse monomeric forms of ETs often have gallic acid in their structures and can be bound together to form oligomers and polymers. Despite the high molecular weight of ETs and thus a limited bio-availability, numerous biological activities have been ascribed to them, such as antioxidant, anti-inflammatory and anticancer properties [2]. These effects are due to the gut microbiota that can convert ETs and ellagic acid to more bio-available metabolites, mainly urolithins, which have free and conjugated forms that are in both the blood and urine [3–7]. However, it is noteworthy that the metabolism and bio-activity of ETs should not be overgeneralized because of their diverse chemical structure, which largely depends on the plant source. For example, a recent study performed by our group showed that the ingestion of defatted raspberry seeds, compared to defatted strawberry seeds, resulted in a lower concentration of their metabolites in the distal intestine, although they contained almost twice as much ETs [8].

Strawberries are abundant in antioxidant compounds, mainly vitamin C, anthocyanins and ETs, which together determine their high in vitro antioxidant capacity [9]. ETs are present in strawberries as monomers and dimers with dimeric agrimoniin found as a predominant ET both in the whole fruits and especially in their pomace, which is a by-product of juice manufacturing [10–13]. Many experiments have shown that strawberry consumption can exert a significant antioxidant effect on the organism; however, a possible contribution of ETs to this action has not been recognized thus far [9, 13].

Additional dietary components of plant origin interacting with the gut microbiota are prebiotic fructo-oligosaccharides (FOS), which cannot be digested by pancreatic enzymes and serve as an energy substrate for specific intestinal bacteria. FOS become more and more popular dietary ingredient that occur naturally in many vegetables, as well as some cereals and fruits. FOS ingestion improves the overall gastrointestinal condition and leads to the formation of preferable intestinal microbiota, among other effects [14]. A recent study performed in our laboratory suggested that dietary FOS can increase the content of nasutin-A, a metabolite of strawberry ETs, in the intestinal digesta of rats. Thus, some beneficial changes in the gut microbiota following simultaneous ingestion of those dietary components as well as a possible increase in ET bio-availability have been suggested [15].

We hypothesized that various classes of strawberry ETs are disparately metabolized in the distal intestine and that the process is modulated by dietary FOS, which in turn can affect the absorption of ET metabolites and the antioxidant status of the organism. Thus, a nutritional experiment on rats was designed to determine the effects of dietary supplementation with FOS and strawberry extracts, containing various proportions of monomeric and dimeric ETs, on the occurrence of selected bacterial populations and the metabolite formation in the distal intestine. Moreover, the effects of the dietary treatments on the plasma concentration of ET metabolites, the plasma antioxidant capacity and the degree of lipid peroxidation in selected tissues (liver, heart and kidney) were investigated.

## Methods

### Preparation of strawberry ET-rich extracts and their analysis

The extracts were obtained from strawberry fruit pomace, a by-product of the manufacture of concentrated strawberry juice (ALPEX Co., Łęczeszyce, Poland), as described by Juśkiewicz et al. [16]. Briefly, the fresh pomace was dried in an industrial vacuum dryer at 70 ± 2 °C and then passed through sieves. The seedless fraction was subjected to a two-stage extraction with a 60 % aqueous solution of acetone. Next, after partial removal of the solvent via distillation, the resultant solutions were transferred onto a column packed with polymeric resin (Amberlite XAD 16, Sigma-Aldrich). The sugars and other water-soluble compounds present in the solutions were eluted from the column with water. Then, monomeric and dimeric ET-rich fractions were desorbed with 10 and 40 % aqueous solution of ethanol, respectively, concentrated to ca. 15 % of dry matter and lyophilized. The methods used to determine the composition of the monomeric ET- and dimeric ET-rich extracts (details in Table 1) are described below.

**Table 1:** Chemical composition of strawberry ET extracts

	Monomeric ET-rich extract (g/100 g)	Dimeric ET-rich extract (g/100 g)
Dry matter	94.31 ± 0.25	91.31 ± 0.05
Ash	0.34 ± 0.02	0.03 ± 0.04
Fat	–	–
Protein	5.62 ± 0.05	1.83 ± 0.03
Other components^a^	0.35 ± 0.01	7.17 ± 0.01
Total polyphenols	88.0 ± 0.1	82.3 ± 0.1
Ellagic acid	0.1 ± 0.0	0.2 ± 0.0
ETs	80.0 ± 0.1	57.3 ± 0.1
Monomers	77.1 ± 0.1	23.3 ± 0.1
Dimers	2.9 ± 0.0	34.0 ± 0.1
Proanthocyanidins	8.1 ± 0.3	23.9 ± 0.2
Flavonols	–	0.9 ± 0.0

The results are expressed as the mean ± SD, *n* = 2

*ETs* ellagitannins

^a^Low molecular carbohydrates and structural components of plant cell walls, including dietary fibre

The basic chemical composition of the extracts was determined according to the official methods of AOAC [17] using the following procedures: 940.26 (dry substance and ash), 920.152 (protein), 930.09 (raw fat) and 985.29 (total dietary fibre).

The concentrations of ETs, ellagic acid, anthocyanins and flavonols were determined in the extracts after their dilution in methanol (1 mg/mL) using HPLC (Knauer Smartline system with photodiode array detector, Berlin, Germany) coupled with a Gemini C18 column (110 Å, 250 × 4.60 mm; 5 μm, Phenomenex, Torrance, USA). Phase A was 0.05 % phosphoric acid in water, phase B was 0.05 % phosphoric acid in 80 % acetonitrile, the flow rate was 1.25 mL/min, the sample volume was 20 μL, and the temperature was 35 °C. Gradient: stabilization for 5 min with 4 % of phase B, 4–15 % B for 5–12.5 min, 15–40 % B for 12.5–42.5 min, 40–50 % B for 42.5–51.8 min, 50–55 % B for 51.8–53.4 min and 4 % B for 53.4–55 min. The following standards were used for the identification of the polyphenols: ellagic acid, flavonols (quercetin-3-O-glucoside, kaempferol-3-O-glucoside, quercetin, kaempferol, tiliroside), pelargonidin-3-O-glucoside (all from Extrasynthese, Genay, France), *p*-coumaric acid (Sigma-Aldrich) and samples of ETs, specifically hexahydroxydiphenoyl-d-glucose and agrimoniin, obtained by semipreparative HPLC as described by Sójka et al. [12]. The absorbance was measured at 280 nm (*p*-coumaric acid, tiliroside, hexahydroxydiphenoyl-d-glucose and agrimoniin), 360 nm (ellagic acid, quercetin, kaempferol and kaempferol glycosides) and 520 nm (anthocyanins).

The concentration of proanthocyanidins in the extracts was determined by the HPLC method after proanthocyanidin breakdown in an acidic environment with an excess of phloroglucinol, according to Kennedy and Jones [18]. The obtained breakdown products were separated using Knauer Smartline chromatograph (Berlin, Germany) equipped with an UV–Vis detector (PDA 280, Knauer, Berlin, Germany) and a fluorescence detector (Shimadzu RF-10Axl, Kyoto, Japan) and coupled with a Gemini C18 column (110 Å, 250 × 4.60 mm; 5 μm, Phenomenex, Torrance, USA). The separation conditions were described by Kosmala et al. [8]. The identification was performed at 280 nm using a UV–Vis detector and the following standards: (−)-epicatechin, (+)-catechin, (−)-epigallocatechin and their respective phloroglucinol adducts. Quantification was conducted by peak areas registered by a fluorescence detector (excitation wavelength 278 nm; emission wavelength 360 nm). Standard curves of (−)-epicatechin and (+)-catechin for terminal units and (−)-epicatechin-phloroglucinol adduct for extender units were used to quantify the breakdown products.

### Feeding experiment

The experiment was conducted on 48 male Wistar rats weighing 257 ± 0.858 g, randomly assigned to one of six groups of eight rats each. The animals were maintained individually in metabolic cages under a stable temperature (21–22 °C), a 12-h light:12-h dark cycle and a ventilation rate of 15 air changes per hour. The rats were used in compliance with the European guidelines for the care and use of laboratory animals, and the animal protocol was approved by the Local Institutional Animal Care and Use Committee (permission no. 32/2012; Olsztyn, Poland). For 4 weeks, the rats had free access to tap water and semipurified diets, which were prepared and then stored at 4 °C in hermetic containers until the end of the experiment. The diets were modifications of a casein diet for laboratory rodents recommended by the American Institute of Nutrition [19] (details in Table 2). Two groups of rats were fed with a control diet containing either cellulose (CEL; 6 % of the diet) or CEL and FOS (3 % of the diet each) as dietary fibre sources (groups C or C + FOS, respectively). Raftilose P95 with a degree of polymerization 2-7 (Beneo-Orafti, Oreye, Belgium) was used in the diets as the FOS source. The other four groups were fed with the above-mentioned diets but also contained either the monomeric ET-rich extract (0.23 % of the diets, groups ME and ME + FOS) or the dimeric ET-rich extract (0.24 % of the diets, groups DE and DE + FOS), which were both added at the expense of corn starch. All experimental diets had a similar content of polyphenols; however, they differed in terms of the content of monomeric and dimeric ETs and proanthocyanidins. The diets fed to the ME and ME + FOS groups had a higher total ET content with a monomer-to-dimer ratio of 96 to 4. The diets fed to the DE and DE + FOS group had a lower total ET content with a monomer-to-dimer ratio of 40 to 60.

**Table 2:** Composition of the group-specific diets

	Group (%)					
	C	C + FOS	ME	ME + FOS	DE	DE + FOS
Casein	14.8	14.8	14.8	14.8	14.8	14.8
DL-methionine	0.2	0.2	0.2	0.2	0.2	0.2
Rapeseed oil	8	8	8	8	8	8
Cellulose	6	3	6	3	6	3
Fructo-oligosaccharides^a^	–	3	–	3	–	3
Corn starch	66.3	66.3	66.07	66.07	66.06	66.06
Monomeric ET-rich extract^2^	–	–	0.23	0.23	–	–
Dimeric ET-rich extract^b^	–	–	–	–	0.24	0.24
Vitamin mix^c^	1	1	1	1	1	1
Mineral mix^c^	3.5	3.5	3.5	3.5	3.5	3.5
Choline chloride	0.2	0.2	0.2	0.2	0.2	0.2
Calculated dietary contents						
Total polyphenols	–	–	0.203	0.203	0.197	0.197
Ellagitannins *(monomer:dimer ratio)*	–	–	0.184 *(96:4)*	0.184 *(96:4)*	0.138 *(40:60)*	0.138 *(40:60)*

*ET* ellagitannins

^a^Raftilose P95 with a degree of polymerization 2-7 (Beneo-Orafti, Oreye, Belgium)

^b^Chemical composition in Table 1

^c^Recommended for the AIN-93 M diet [19]

### Sample collection and basic analyses

At the termination of the experiment, the rats were weighed and anesthetized with sodium pentobarbital (50 mg/kg body weight). After a laparotomy, blood samples were collected from the caudal vein and stored in tubes containing heparin, and the intestinal segments (small intestine, caecum and colon) and internal organs (heart, kidneys and liver) were removed and weighed. The blood was then centrifuged for 15 min at 380×*g*, and the obtained plasma was stored at −20 °C until analyses.

Samples of the ileal, caecal and colonic digesta were collected, and the pH was immediately measured using a microelectrode and a pH/ION meter (model 301; Hanna Instruments, Vila do Conde, Portugal). In the fresh caecal digesta, the dry matter was determined at 105 °C, whereas the ammonia concentration was determined by the microdiffusion method in Conway’s dishes. After storage of the caecal digesta at −70 °C, the short-chain fatty acid (SCFA) concentrations were measured using gas chromatography (Shimadzu GC-2010, Kyoto, Japan) and a capillary column (SGE BP21, 30 m × 0.53 mm; SGE Europe Ltd., Milton Keynes, UK) as previously described [8].

In the blood plasma, the antioxidant capacity of water-soluble and lipid-soluble substances (ACW and ACL, respectively) was determined by a photochemiluminescence detection method using a Photochem and respective kits (ACW-Kit and ACL-Kit, Analytik Jena AG, Germany). In the photochemiluminescence assay, the generation of free radicals was partially eliminated through reactions with antioxidants present in the plasma samples, and the remaining radicals were quantified by luminescence generation. Ascorbate and Trolox calibration curves were used to evaluate ACW and ACL, respectively.

Thiobarbituric acid-reactive substances (TBARS) were determined in the heart, kidney and liver tissue after their storage at −70 °C. A procedure developed by Botsoglou et al. [20] was used in the assay, and the TBARS contents were determined spectrophotometrically at 532 nm and expressed in µg malondialdehyde per g of tissue.

### Determining bacterial counts

The caecal digesta was weighed and subjected to a bacterial DNA isolation procedure using a bead-beating method according to the protocol of the GeneMATRIX Stool DNA Purification Kit (Eurx, Gdańsk, Poland). Quantitative real-time polymerase chain reaction (PCR) was run in duplicate. The reactions were conducted in an iQ5 real-time PCR system (Bio-Rad, Warsaw, Poland) in a total volume of 25 µL consisting of 12.5 µL of SYBR^®^ Green JumpStart™ Taq ReadyMix™ (Sigma-Aldrich), 200 µM of each respective primer, 1 µL of tenfold diluted DNA and PCR-grade water (Sigma-Aldrich). The amplification was based on an initial denaturation at 95 °C for 3 min, followed by 35 cycles of denaturation at 95 °C for 20 s, annealing at a primer-specific temperature for 30 s and a final cycle at 72 °C for 30 s. Primer sequences and annealing temperatures are available upon request. After completion of the amplifications, a melting curve was prepared to confirm the specificity of PCR products. The construction of the standard curve for each real-time PCR analysis was based on bacterial strains from our own culture collection and from the German Collection of Microorganisms and Cell Cultures. Each bacterial culture was separately cultivated in appropriate conditions (details available upon request), and the cell count was determined using 4′,6-diamidino-phenylindole (Sigma-Aldrich) with a procedure described elsewhere [21]. Two millilitres of each culture was centrifuged (5 min, 10,000×*g*), washed with sterile PBS (pH 7.4) and then centrifuged again. The cell pellets were combined and mixed with 0.1 g of the autoclaved caecal digesta, and bacterial DNA was isolated with the above-mentioned method. Isolated DNA was serially diluted and used for the construction of the standard curve for each primer pair tested. The data were analysed using iQ5 Optical System Software (version 2.0) and expressed as the cell count per g of the caecal digesta.

### Quantification of ellagic acid and ET metabolites

Ellagic acid concentration was determined in the caecal digesta after their hydrolysis with trifluoroacetic acid. The digesta (0.2 mg) was mixed with 70 % glycerol (0.5 mL) and 75 µL of trifluoroacetic acid and incubated at 95 °C for 18 h. Afterwards, the sample was cooled and extracted 3 times using 1.5 mL of methanol in an ultrasonic bath. After each extraction, the sample was centrifuged (3 min, 10,000×*g*), and the supernatant was collected in a volumetric flask and filled up with methanol. Ellagic acid was then determined using HPLC (Knauer Smartline system with photodiode array detector, Berlin, Germany) coupled with a Gemini C18 column (110 Å, 250 × 4.60 mm; 5 μm, Phenomenex, Torrance, USA). Phase A was 0.05 % phosphoric acid in water, phase B was 0.05 % phosphoric acid in 80 % acetonitrile, the flow rate was 1.25 mL/min, the sample volume was 20 μL, and the temperature was 35 °C. The gradient was as follows: 10–25 % B for 0–10 min, 25–40 % B for 10–20 min, 40–80 % B for 20–25 min, 80 % B for 25–30 min, 80–10 % B for 30–32 min, 10 % B for 32–40 min. The identification and quantification were performed at 360 nm with ellagic acid as a standard.

The concentration of ET metabolites was determined in the caecal digesta and plasma. A frozen sample of the digesta (0.5–1 g) was mixed with acetone (2 mL), sonicated for 10 min and centrifuged (5 min, 10,000×*g*), and then, the supernatant was collected in a test tube. The procedure was repeated twice with 2 and 1 mL of 70 % acetone. After collection of the supernatant, the extract was concentrated using a vacuum concentrator (ScanSpeed 40, Labogene, Denmark) and then dissolved in methanol (1 mL). ET metabolites were then determined using HPLC (Knauer Smartline system with photodiode array detector, Berlin, Germany) coupled with a Gemini C18 column (110 Å, 250 × 4.60 mm; 5 μm, Phenomenex, Torrance, USA). Separation conditions were the same as those used in the determination of ETs in dietary extracts. ET metabolites were identified by comparison of UV spectra with the available literature data [22] and additionally confirmed by a MS/MS method described below. A plasma sample (0.5 mL) was mixed with acetone (1 mL), sonicated for 10 min and centrifuged (5 min, 10,000×*g*), and then, the supernatant was collected in a test tube. The procedure was repeated, and both supernatants were collected in a test tube and concentrated using a vacuum concentrator (ScanSpeed 40, Labogene, Denmark). Next, the concentrated sample was dissolved in methanol (200 µL) and analysed by HPLC–ESI–MS using a Dionex UltiMate 3000 UHPLC and a Thermo Scientific Q Exactive series quadrupole ion trap mass spectrometer. ET metabolites were separated using a Kinetex C18 column (110 Å, 150 × 2.1 mm; 2.6 μm, Phenomenex, Torrance, USA) and a binary gradient of 0.1 % formic acid in water (phase A) and 0.1 % formic acid in acetonitrile (phase B) at a flow rate of 0.5 mL/min, as follows: stabilization for 1.44 min with 5 % B, 5–15 % B for 1.44–2.98 min, 15–40 % B for 2.98–10.1 min, 40–73 % B for 10.1–11.5 min, 73 % B for 11.55–12.7 min, 73–5 % B for 12.7–13.28 min, 5 % B for 13.28–18 min. The MS analysis was performed in negative ion mode under the following conditions: capillary voltage at +4 kV, sheath gas pressure at 75 arbitrary units, auxiliary gas at 17 arbitrary units and scan range 120–1200 *m/z*. Urolithin-A isolated from human urine by semipreparative HPLC was used as a standard for the quantification of ET metabolites. The detailed procedure of urolithin-A isolation is described elsewhere [15].

### Statistical analysis

The results are expressed as the mean ± SEM except for the chemical composition of the strawberry extracts, which is expressed as the mean ± SD. A 2-factor analysis of variance (ANOVA) was used to determine the effect of the extract additions (none, monomeric ET- or dimeric ET-rich extract) and the FOS addition and the interaction between these two factors (Extract × FOS). If the analysis revealed a significant interaction or both effects or the extract additions had a significant effect (*P* ≤ 0.05), the differences among the respective treatment groups were then determined with Duncan’s post hoc test at *P* ≤ 0.05. If ANOVA assumptions were violated, the Kruskal–Wallis one-way ANOVA by ranks was used followed by Dunn’s post hoc test (*P* ≤ 0.05). The statistical analysis was performed using STATISTICA software, version 10.0 (StatSoft Corp., Kraków, Poland).

## Results

After 4 weeks of experimental feeding, the diet intake and body weight gain did not differ among the groups (data not shown, *P* > 0.05). The mass of the small intestine with contents relative to body weight was increased by the FOS addition (*P* < 0.005), whereas the pH value of the ileal digesta was decreased (*P* < 0.001, Table 3). Moreover, the caecal tissue mass relative to body weight and the caecal digesta mass relative to the tissue mass were increased by dietary FOS (*P* < 0.001 and *P* < 0.01, respectively), whereas the pH value and the ammonia concentration in the caecal digesta were decreased (*P* < 0.001 and *P* < 0.005, respectively; Table 3). An interaction effect between the extract and the FOS addition was also observed in examining the caecal tissue mass (*P* = 0.05); however, no additional relationships in Duncan’s post hoc test were noted. The dry matter of the caecal digesta was affected by the extract addition (*P* < 0.01), and an interaction between this dietary factor and the FOS addition was also noted (*P* < 0.05). Thus, the digesta dry matter was higher in the ME + FOS and DE group than in the C, C + FOS and DE + FOS groups (*P* ≤ 0.05). Moreover, the acetate and total SCFA concentrations in the caecal digesta were affected both by the extract and FOS addition (*P* < 0.05 and P < 0.001, respectively) as well as their interaction (*P* < 0.05). The caecal acetate and total SCFA concentrations were higher in the FOS groups than in the C, ME and DE groups, except for the DE + FOS group in which the concentrations were the same as those in the later groups (*P* ≤ 0.05). The caecal propionate concentration was affected by the extract addition (*P* < 0.001), and its level was lower in the ME + FOS and DE + FOS group than in the C + FOS group (*P* ≤ 0.05). The caecal butyrate concentration was increased by dietary FOS (*P* < 0.005). Moreover, the extract and FOS addition decreased the concentration of putrefactive SCFAs (*P* < 0.05 and *P* < 0.001, respectively) calculated as the sum of isobutyrate, isovalerate and valerate in the caecal digesta. The lowest concentration of putrefactive SCFAs was in the ME + FOS group, and the concentrations were significantly higher in the C + FOS and DE + FOS groups. The highest concentration of SCFAs was in the C group (*P* ≤ 0.05).

**Table 3:** Mass, pH value, dry matter, ammonia and SCFA concentrations in the intestinal digesta of rats fed diets containing monomeric ET- or dimeric ET-rich extract without or with FOS for 4 weeks

	Group^1^						2 Factor ANOVA *P* values		
	C	C + FOS	ME	ME + FOS	DE	DE + FOS	Extract	FOS	Extract × FOS
Small intestine									
Mass with contents^2^	1.87 ± 0.047	1.97 ± 0.045	1.84 ± 0.032	1.97 ± 0.043	1.88 ± 0.040	1.98 ± 0.041	NS	<0.005	NS
pH of ileal digesta	7.51 ± 0.135	6.64 ± 0.107	7.30 ± 0.106	6.85 ± 0.069	7.32 ± 0.142	6.79 ± 0.100	NS	<0.001	NS
Caecum									
Tissue mass^2^	0.170^b^ ± 0.006	0.218^a^ ± 0.006	0.164^b^ ± 0.005	0.218^a^ ± 0.005	0.180^b^ ± 0.006	0.205^a^ ± 0.008	NS	<0.001	=0.05
Digesta mass^3^	3.04 ± 0.219	3.96 ± 0.137	3.41 ± 0.284	3.56 ± 0.274	3.03 ± 0.063	3.51 ± 0.296	NS	<0.01	NS
pH of digesta	7.57 ± 0.097	6.82 ± 0.070	7.47 ± 0.104	6.45 ± 0.120	7.41 ± 0.093	6.82 ± 0.083	NS	<0.001	NS
Dry matter, %	23.9^b^ ± 0.815	23.8^b^ ± 0.660	25.2^ab^ ± 0.221	26.5^a^ ± 0.696	26.4^a^ ± 0.470	24.4^b^ ± 0.790	<0.01	NS	<0.05
NH_3_, mg/g	0.259 ± 0.010	0.209 ± 0.009	0.222 ± 0.007	0.225 ± 0.013	0.238 ± 0.012	0.198 ± 0.018	NS	<0.005	NS
SCFAs, μmol/g	97.3^b^ ± 2.98	127^a^ ± 5.07	106^b^ ± 5.18	122^a^ ± 6.3	99.2^b^ ± 5.00	103^b^ ± 3.87	<0.05	<0.001	<0.05
Acetate	65.2^b^ ± 1.79	90.5^a^ ± 4.29	74.7^b^ ± 3.22	91.1^a^ ± 7.24	70.7^b^ ± 3.85	71.7^b^ ± 4.44	<0.05	<0.001	<0.05
Propionate	16.8^ab^ ± 0.763	19.1^a^ ± 1.02	15.1^bc^ ± 0.856	14.7^bc^ ± 1.25	14.1^bc^ ± 1.04	12.7^c^ ± 0.404	<0.001	NS	NS
Butyrate	9.55 ± 0.90	13.27 ± 1.72	11.29 ± 1.64	13.83 ± 2.39	9.39 ± 1.00	14.56 ± 1.30	NS	<0.005	NS
Putrefactive SCFAs^4^	5.75^a^ ± 0.321	3.91^b^ ± 0.470	4.60^ab^ ± 0.233	2.55^c^ ± 0.267	4.96^ab^ ± 0.603	4.05^b^ ± 0.549	<0.05	<0.001	NS

Values are mean ± SEM, *n* = 8. Means within a column without a common letter differ, *P* ≤ 0.05 (Duncan’s post hoc test). *CEL* cellulose, *ETs* ellagitannins, *FOS* fructo-oligosaccharides, *NS* non-significant data, *P* > 0.05

^1^C, control fed a diet with CEL as a sole source of dietary fibre; C + FOS, control fed a diet with FOS added at the expense of CEL; ME, fed a diet supplemented with a monomeric ET-rich extract; ME + FOS, fed a diet containing FOS and supplemented with a monomeric ET-rich extract; DE, fed a diet supplemented with a dimeric ET-rich extract; DE + FOS, fed a diet containing FOS and supplemented with a dimeric ET-rich extract

^2^g/100 g body weight

^3^g/g caecal or colonic tissue

^4^Calculated as the sum of isobutyrate, isovalerate and valerate

The total and selected bacterial counts in the caecal digesta are shown in Table 4. The extract addition, the FOS addition and their interaction significantly affected the total bacterial count (all *P* < 0.001), which was the highest in the C + FOS group, significantly lower in the DE group and the lowest in the ME + FOS group (all *P* ≤ 0.05). The count of *Atopobium* genus was affected by the extract addition (*P* < 0.001), and an interaction between both dietary factors was noted (*P* < 0.005). The lowest *Atopobium* counts were in the ME + FOS group, and they were significantly increased in the ME group (*P* ≤ 0.05) but not to the highest level observed in the C + FOS and DE + FOS groups (*P* ≤ 0.05). The count of *Bifidobacterium* genus was affected by the extract addition and by dietary FOS (both *P* < 0.001) as well as their interaction (*P* < 0.005). The *Bifidobacterium* counts were the highest in the C + FOS group and the lowest in both ME groups. In the DE group, the *Bifidobacterium* counts were also relatively low; however, the FOS addition increased them significantly (*P* ≤ 0.05), which was not observed in the ME groups. Furthermore, the count of *Enterococcus* genus was affected by both dietary factors at *P* < 0.001. The lowest *Enterococcus* counts were noted in the ME + FOS group, and they were significantly higher in the DE + FOS group, whereas the highest values were noted in the C group (all *P* ≤ 0.05). The count of *Lactobacillus* genus was increased by the FOS addition (*P* < 0.001), but the extract addition also had a significant effect on it (*P* < 0.05). Thus, the *Lactobacillus* counts did not differ between the ME + FOS group and the ME group, whereas they were increased in the C and DE group compared to those of the C + FOS and DE + FOS group, respectively. Moreover, the count of *Bacteroides*-*Prevotella*-*Porphyromonas* was affected by both tested factors and their interaction (all *P* < 0.001), and thus, it was lower in the ME + FOS group than in the other groups (*P* ≤ 0.05). The *Clostridium leptum* group was significantly affected by both tested factors and their interaction (all at *P* < 0.001), and its count was lower in the ME + FOS group than in the other groups (*P* ≤ 0.05).

**Table 4:** Total and selected bacterial counts quantified by real-time PCR in the caecal digesta of rats fed diets containing monomeric ET- or dimeric ET-rich extract without or with FOS for 4 weeks

	Group^1^ (log cells/g)						2 Factor ANOVA *P* values		
	C	C + FOS	ME	ME + FOS	DE	DE + FOS	Extract	FOS	Extract × FOS
Total bacteria	10.5^ab^ ± 0.079	10.6^a^ ± 0.084	10.4^ab^ ± 0.055	8.52^c^ ± 0.104	10.3^b^ ± 0.071	10.5^ab^ ± 0.050	<0.001	<0.001	<0.001
Genus									
*Atopobium*	9.36^ab^ ± 0.182	9.56^a^ ± 0.109	8.99^b^ ± 0.059	8.23^c^ ± 0.088	9.11^ab^ ± 0.057	9.54^a^ ± 0.305	<0.001	NS	<0.005
*Bifidobacterium*	8.05^c^ ± 0.503	10.3^a^ ± 0.369	6.83^d^ ± 0.210	6.40^d^ ± 0.399	7.35 ^cd^ ± 0.299	9.18^b^ ± 0.400	<0.001	<0.001	<0.005
*Enterococcus*	6.80^a^ ± 0.359	6.06^ab^ ± 0.233	6.10^ab^ ± 0.158	4.46^c^ ± 0.100	6.50^ab^ ± 0.314	5.90^b^ ± 0.153	<0.001	<0.001	NS
*Lactobacillus*	5.45^bc^ ± 0.354	6.92^a^ ± 0.408	4.96^c^ ± 0.262	5.41^bc^ ± 0.398	4.68^c^ ± 0.358	6.11^ab^ ± 0.174	<0.05	<0.001	NS
Group									
*Bacteroides*-*Prevotella*-*Porphyromonas*	9.62^a^ ± 0.139	9.75^a^ ± 0.123	9.64^a^ ± 0.068	7.73^b^ ± 0.080	9.54^a^ ± 0.109	9.40^a^ ± 0.172	<0.001	<0.001	<0.001
*Clostridium leptum*	7.83^a^ ± 0.082	7.70^a^ ± 0.308	8.07^a^ ± 0.149	6.10^b^ ± 0.283	7.74^a^ ± 0.068	7.78^a^ ± 0.277	<0.005	<0.001	<0.001

Values are mean ± SEM, *n* = 6. Means within a column without a common letter differ, *P* ≤ 0.05 (Duncan’s post hoc test). CEL, cellulose; ETs, ellagitannins; FOS, fructo-oligosaccharides; NS, non-significant data, *P* > 0.05; PCR, polymerase chain reaction

^1^C, control fed a diet with CEL as a sole source of dietary fibre; C + FOS, control fed a diet with FOS added at the expense of CEL; ME, fed a diet supplemented with a monomeric ET-rich extract; ME + FOS, fed a diet containing FOS and supplemented with a monomeric ET-rich extract; DE, fed a diet supplemented with a dimeric ET-rich extract; DE + FOS, fed a diet containing FOS and supplemented with a dimeric ET-rich extract

The ellagic acid determined after hydrolysis of the caecal digesta was absent in both C groups, whereas in the other groups, it was present at various concentrations (*P* < 0.001). The ellagic acid concentration was lower in the DE group than in the ME + FOS and DE + FOS groups (*P* ≤ 0.05, Table 5). ET-specific metabolites were detected in the caecal digesta and blood plasma of the ME and DE groups, but they were not present at all in the C groups. The total concentration of ET metabolites in the caecal digesta was higher in the ME and ME + FOS groups than in the DE and DE + FOS groups. Nasutin-A was the main ET metabolite found in the caecal digesta, and its concentration was higher in the ME groups than in the DE groups (*P* ≤ 0.05). In the caecal digesta, a relatively low concentration of urolithin-A was also found, but only in the ME + FOS group. Additionally, isonasutin-A-glucuronide was present in the caecal digesta of all ME and DE groups, and its concentration was higher in the ME + FOS group than in the ME group (*P* ≤ 0.05). The total plasma concentration of ET metabolites was higher in the ME + FOS and DE + FOS groups than in the ME group. Urolithin-A was the main plasma metabolite found in the ME + FOS, DE and DE + FOS groups, whereas in the ME group, it was not present. The plasma urolithin-A concentration was higher in the DE + FOS group than in the DE group (*P* ≤ 0.05). Additionally, isonasutin-A-glucuronide and nasutin-A-glucuronide were identified in the blood plasma. The plasma isonasutin-A-glucuronide concentration was on a similar level in both DE groups and in the ME + FOS group, whereas in the ME group, it was not detected. The plasma nasutin-A-glucuronide concentration was the highest in the ME group and was slightly and significantly lower in the ME + FOS and DE groups, respectively (*P* > 0.05 and *P* ≤ 0.05, respectively). In the DE + FOS group, the metabolite was absent.

**Table 5:** Concentration of ellagic acid in hydrolysed caecal digesta and ET metabolites in the caecal digesta and plasma of rats fed diets containing monomeric ET- or dimeric ET-rich extract without or with FOS for 4 weeks

	Group^1^				Kruskal–Wallis ANOVA *P* value
	ME	ME + FOS	DE	DE + FOS	
Caecal digesta					
Ellagic acid, mg/g	1.95^ab^ ± 0.058	2.41^a^ ± 0.016	1.61^b^ ± 0.024	2.04^a^ ± 0.065	<0.001
Total metabolites, µg/g	67.8^ab^ ± 10.7	89.5^a^ ± 15.0	13.0^c^ ± 0.98	18.0^bc^ ± 3.33	<0.001
Nasutin-A^2^, µg/g	62.7^a^ ± 19.5	79.2^a^ ± 15.3	6.28^b^ ± 1.37	10.3^b^ ± 3.82	<0.001
Isonasutin-A-glucuronide^3^, µg/g	5.12^b^ ± 0.363	9.19^a^ ± 1.09	6.68^ab^ ± 0.568	7.76^ab^ ± 0.860	<0.05
Urolithin-A^4^, µg/g	ND	1.10 ± 0.384	ND	ND	–
Plasma					
Total metabolites, ng/mL	8.13^b^ ± 2.12	248^a^ ± 43.8	146^ab^ ± 19.2	281^a^ ± 47.5	<0.001
Urolithin-A^4^, ng/mL	ND	235^ab^ ± 44.0	136^b^ ± 19.1	271^a^ ± 47.9	<0.01
Isonasutin-A-glucuronide^3^, ng/mL	ND	10.1 ± 1.25	10.1 ± 0.834	10.8 ± 1.50	NS
Nasutin-A-glucuronide^5^, ng/mL	8.13^a^ ± 2.12	2.65^ab^ ± 1.75	0.283^b^ ± 0.283	ND	<0.005

Values are mean ± SEM, *n* = 8. Means within a column without a common letter differ, *P* ≤ 0.05 (Dunn’s post hoc test). *ETs* ellagitannins, *FOS* fructo-oligosaccharides, *ND* not detected, *NS* non-significant data, *P* > 0.05

^1^ME, fed a diet supplemented with a monomeric ET-rich extract; ME + FOS, fed a diet containing FOS and supplemented with a monomeric ET-rich extract; DE, fed a diet supplemented with a dimeric ET-rich extract; DE + FOS, fed a diet containing FOS and supplemented with a dimeric ET-rich extract

^2^HPLC retention time (min) 10.08; MS [M–H]^−^ 269; MS/MS fragments 113, 85.03; UV spectra (nm) 227, 245, 284, 323, 389

^3^HPLC retention time (min) 8.08; MS [M–H]^−^ 445; MS/MS fragments 269.01, 113, 85.03; UV spectra (nm) 218, 264, 332

^4^HPLC retention time (min) 9.54; MS [M–H]^−^ 227; MS/MS fragments 113.02, 85.03; UV spectra (nm) 197, 218, 280, 307, 355

^5^HPLC retention time (min) 6.99; MS [M–H]^−^ 445; MS/MS fragments 269.01, 113, 85.03; UV spectra (nm) 222, 279, 315, 367, 379

**Fig. 1:**
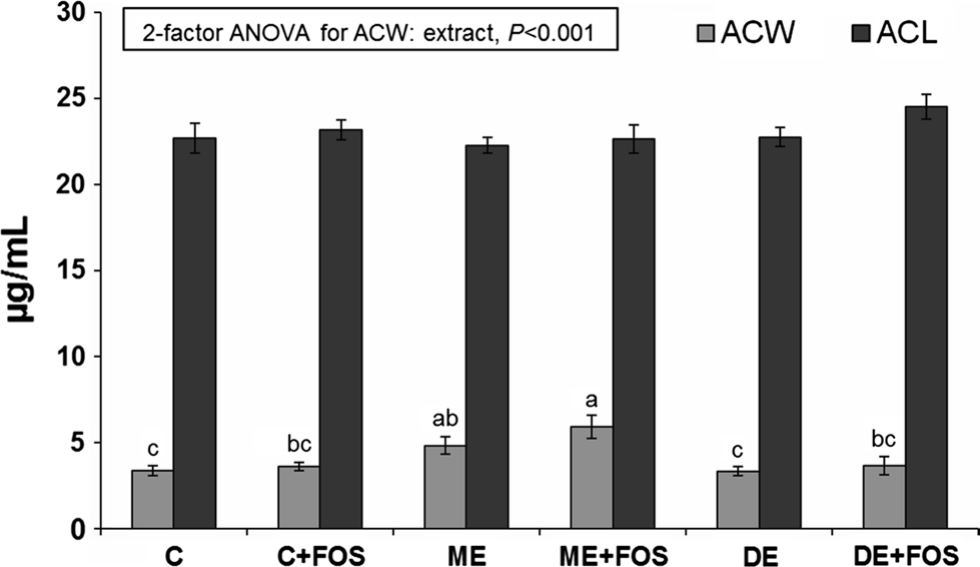
Plasma ACW and ACL in rats fed diets containing monomeric ET- or dimeric ET-rich extract without or with FOS for 4 weeks. Values are mean ± SEM, *n* = 8. Means without a common letter differ, *P* ≤ 0.05 (Duncan’s post hoc test). *ACL* antioxidant capacity of lipid-soluble substances, *ACW* antioxidant capacity of water-soluble substances, *CEL* cellulose, *ETs* ellagitannins, *FOS* fructo-oligosaccharides. *C* control fed a diet with CEL as a sole source of dietary fibre; *C + FOS* control fed a diet with FOS added at the expense of CEL; *ME* fed a diet supplemented with a monomeric ET-rich extract; *ME + FOS* fed a diet containing FOS and supplemented with a monomeric ET-rich extract; *DE* fed a diet supplemented with a dimeric ET-rich extract; *DE + FOS* fed a diet containing FOS and supplemented with a dimeric ET-rich extract

**Fig. 2:**
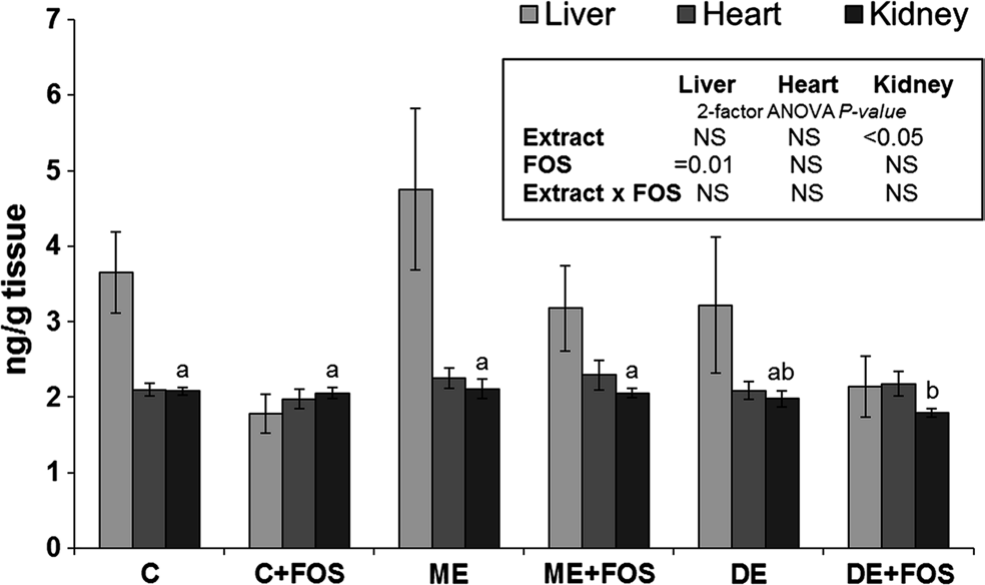
TBARS in the liver, heart and kidney of rats fed diets containing monomeric ET- or dimeric ET-rich extract without or with FOS for 4 weeks. Values are mean ± SEM, *n* = 8. Means without a common letter differ, *P* ≤ 0.05 (Duncan’s post hoc test). *CEL* cellulose, *ETs* ellagitannins, *FOS* fructo-oligosaccharides, *NS* non-significant data, *P* > 0.05; *TBARS* thiobarbituric acid-reactive substances. *C* control fed a diet with CEL as a sole source of dietary fibre, *C* *+ FOS* control fed a diet with FOS added at the expense of CEL, *ME* fed a diet supplemented with a monomeric ET-rich extract; *ME + FOS* fed a diet containing FOS and supplemented with a monomeric ET-rich extract; *DE* fed a diet supplemented with a dimeric ET-rich extract; *DE + FOS* fed a diet containing FOS and supplemented with a dimeric ET-rich extract

The plasma ACW concentration was affected by the extract addition (*P* < 0.001), and it was the highest in the ME + FOS group (*P* ≤ 0.05), slightly lower in the ME group (*P* > 0.05) and significantly lower in the other groups (*P* ≤ 0.05, Fig. 1). The TBARS contents in selected tissues are depicted in Fig. 2. The liver TBARS contents were decreased by dietary FOS (*P* = 0.01), whereas the kidney TBARS contents were affected by the extract addition (*P* < 0.05). Compared to all C and ME groups, the kidney TBARS contents were slightly and significantly lower in the DE and DE + FOS groups, respectively (*P* > 0.05 and *P* ≤ 0.05, respectively).

## Discussion

ETs and ellagic acid are present in high quantities in some fruits, such as pomegranates, blackberries, strawberries, raspberries and cloudberries, as well as in some nuts, such as almonds and walnuts [2, 9]. In strawberries, ETs together with unbounded ellagic acid are thought to be the second largest class of polyphenols after anthocyanins, whereas proanthocyanidins seem to be quantitatively less important [9]. However, a study of Buendía et al. [23] clearly showed that the content of proanthocyanidins can be even higher than ETs and anthocyanins. In strawberry pomace, both high contents of ETs and proanthocyanidins have been found, whereas anthocyanins were minor components, which is most likely due to their easier transfer from the fruit to the juice during industrial pressing [12]. In this study, two components were extracted from strawberry pomace, which contained >82 % polyphenols with ETs as their predominant class (Table 1 **)**. The monomeric ET-rich extract had a higher total content of ETs than the dimeric ET-rich extract (80 vs. 57/100 g extract), whereas the latter had a higher content of proanthocyanidins (24 vs. 8/100 g extract) and contained significant amounts of monomeric ETs (23/100 g extract). All of the obtained differences were due to fractionation of the extracted polyphenols on a synthetic resin.

FOS as a part of dietary fibre occur naturally in many plants, among which tubers of Jerusalem artichoke and chicory root are some of the most abundant sources. A typical intestinal change was observed in the present study following the FOS ingestion, such as acidification of the digesta and an increase in the digesta fresh weight and in total and individual SCFA concentrations, including butyrate concentration [14, 24]. The FOS supplementation also limited putrefaction in the caecum as displayed by the decreased ammonia and putrefactive SCFA concentrations. All of the aforementioned changes are considered beneficial for the organism. For example, butyrate is recognized as a preferred substrate for colonocytes, and it appears to protect against colorectal cancer, whereas ammonia can induce cancerous cell growth [14, 25, 26]. In this study, the FOS supplementation also stimulated lactic acid bacteria and bifidobacteria growth, and these strains can exert probiotic effects [27]. It is worth noting, however, that dietary extracts limited those beneficial changes to some extent, such as the production of SCFAs, and its total concentration did not increase when FOS was added to the diet together with the dimeric ET-rich extract. Moreover, the growth of bifidobacteria and lactic acid bacteria were inhibited by the monomeric ET-rich extract which confirms the antibacterial activity of ETs established in vitro [2, 28]. It has been suggested that ETs can interact with bacterial enzymes and damage bacterial cell walls and membranes [28]. While the available literature data are consistent in terms of the inhibitory effect of ETs on pathogenic bacteria, their effect on probiotic bacteria is not fully recognized. Larrosa et al. [29] showed that the ingestion of ETs and their metabolites can increase *Bifidobacterium* and *Lactobacillus* counts in rat faeces, whereas in human subjects, Bialonska et al. [30] did not find such overall increases; however, the growth of some individual species was enhanced or even inhibited. Surprisingly, in this study, especially strong antibacterial activity was noted in the caecum of rats fed the monomeric ET-rich extract and FOS, in which both the total bacterial population and a majority of individual populations were reduced by at least one order of magnitude (group ME + FOS vs. groups C and C + FOS, Table 4).

Generally, the mechanism of ET breakdown within the gastrointestinal tract is believed to depend on two main factors. First, the intestinal pH can directly hydrolyse ETs, and second, the microbiota can convert ETs and ellagic acid to more bio-available compounds, such as urolithins [2–5, 31, 32]. Importantly, two strains of the *Gordonibacter* genus able to metabolize ellagic acid to urolithins have been recently identified in human faeces [33]. In this case, however, the ET metabolism may be a bacterial defence mechanism against the antibacterial activity of ETs. A lack of trihydroxybenzoyl groups in most ET metabolites confirms this supposition because their presence in ET molecules seems to determine their antibacterial power [28]. Nevertheless, in this study, although up to 1.84 mg ETs were present in a gram of experimental diets, the total concentration of ET metabolites in the caecum did not exceed 90 µg per g digesta. Additionally, the experimental diets contained only traces of unbounded ellagic acid (≤4.8 µg/g diet), whereas their large quantities were released from ET molecules after hydrolysis of the caecal digesta (≥1.6 mg/g digesta). Because the caecum is the main site of intestinal fermentation in rats, the obtained results indicate a very limited breakdown of ETs. This finding is in agreement with Aguilera-Carbo et al. [1] who emphasized difficulties in the biodegradation of ETs. Considering all of the aforementioned, we suggest that the initial release of ellagic acid induced by intestinal pH may be the rate-limiting step in the bacterial metabolism of ETs. Furthermore, in this study, the total concentration of ET metabolites in the caecal digesta was approximately five times higher in the ME and ME + FOS groups than in the respective DE groups. Additionally, nasutin-A was the main caecal metabolite in the ME groups, in which its presence was more than six times greater than that in the DE groups. This result suggests that monomeric ETs are more prone to the intestinal breakdown than dimeric ETs. However, the magnitude of the difference is not as large because the diets fed to the ME groups contained 25 % more ETs than those fed to the DE groups. Nevertheless, this evidence is in agreement with the findings of our previous study, in which a less polymerized structure of strawberry ETs than that of raspberry ETs resulted in a higher caecal concentration of their metabolites [8]. Furthermore, the occurrence of nasutins in animal kingdom has already been reported by Moore [34] in the early 1960s. The present study and our previous studies [8, 15] suggest that nasutin-A is the predominant caecal metabolite of strawberry ETs in rats. However, this finding seems to contradict the other available reports, in which urolithins were found as the only bacterial metabolites of ETs both in rats and humans [3–5, 32]. We suggest two main reasons that can be responsible for this disagreement. First are structural differences between strawberry ETs and pomegranate ETs, which were mainly tested in other studies, and may significantly affect microbial metabolism. Second is the analysis of caecal digesta instead of faeces, as it took place in other animal experiments as well as in human studies; thus, the time for interaction between strawberry ETs and the gut microbiota was much shorter.

In the present study, the absorption of ET metabolites into the bloodstream was also very limited, and although their micrograms were present in a gram of the caecal digesta, the total plasma concentration of ET metabolites did not exceed 300 ng/mL. Moreover, urolithin-A was the predominant plasma metabolite in most groups, and only small amounts of nasutin glucuronide derivatives were determined. This finding suggests that nasutin-A found as the main metabolite in the caecal digesta was barely, but to some extent, absorbed and might have been further metabolized and absorbed in the colon. This situation might have taken place especially in the DE groups, in which the total plasma concentration of ET metabolites was as high as in the respective ME groups. Surprisingly, urolithin-A was not present at all in the plasma of the ME group, and only traces of nasutin-A-glucuronide were determined there. Perhaps colonic bacteria were somehow able to transform monomeric ETs to much more simple compounds, such as phenolic acids and phloroglucinol, which are recognized to be the main polyphenol metabolites of gut microbiota [35]. However, the observed strong antibacterial activity of the monomeric ET-rich extract and FOS was probably enough to slow the bacterial breakdown, so we could determine more ET metabolites both in the caecal digesta and plasma of the ME + FOS group compared to the ME group. Moreover, the plasma urolithin-A was found in only an unconjugated form, whereas both the plasma nasutin-A and insonasutin-A were glucuronidated to increase their water solubility and to facilitate their excretion, as is the case for many other polyphenols [36].

Oxidative stress can cause cell damage and underlies various chronic diseases and ageing; thus, dietary antioxidants and their bio-availability are of interest [37]. ETs have high in vitro antioxidant activity; however, the results of in vivo studies are equivocal. An early study by Cerdá et al. [4] indicated that urolithins found in the plasma and urine of volunteers drinking pomegranate juice did not exert significant antioxidant activity. However, a more recent study by Hassimotto and Lajolo [38] showed that although blackberry ETs gavaged to rats did not increase the plasma antioxidant capacity, they were able to reduce lipid peroxidation determined by TBARS assay in select tissues (kidney, liver and brain). Another study on rats with gastric ulcers showed that an ellagitannin-rich fraction from *Eucalyptus citriodora* exerted dose-dependent antioxidant effects on the stomach tissue [39]. In this study, both dietary extracts beneficially affected the antioxidant status of rats if they were supplemented together with FOS, which is, to some extent, consistent with the FOS-induced increase in plasma ET metabolites. The monomeric ET-rich extract increased the plasma ACW concentration, whereas the dimeric ET-rich extract decreased the content of TBARS in kidney tissue. The reasons for these differences are not clear; however, the antioxidant balance of an organism depends on many interdependent antioxidants, such as glutathione, uric acid, antioxidant vitamins and enzymes [37].

In conclusion, although ETs of the monomeric ET-rich extract are more prone to the intestinal breakdown than those of the dimeric ET-rich extract, the overall bacterial metabolism of strawberry ETs and absorption of their metabolites into the bloodstream is very limited. Moreover, we suggest that the initial release of ellagic acid induced by intestinal pH may be the rate-limiting step in the bacterial metabolism of ETs. A simultaneous inclusion of the monomeric ET-rich extract and FOS in the diet evokes strong antibacterial activity in the caecum of rats, and it increases the intestinal absorption of ET metabolites due to their reduced intestinal breakdown. Strawberry ET-rich extracts can moderately improve the antioxidant status of rats by increasing the plasma ACW level if the monomeric ET-rich extract is included in the diet or by decreasing lipid peroxidation in the kidney if the dimeric ET-rich extract and FOS are included in the diet.
